# Forward geometric model prediction of a 6-RSU parallel manipulator using a modified NARX Bayesian neural network

**DOI:** 10.1016/j.heliyon.2024.e41047

**Published:** 2024-12-09

**Authors:** Alaa Aldeen Joumah, Assef Jafar, Chadi Albitar

**Affiliations:** Higher Institute for Applied Sciences and Technology (HIAST), Damascus, P.O.Box 31983, Syria

**Keywords:** Parallel manipulator, Forward geometric model, Bayesian neural network, Uncertainty, NARX, Variational inference

## Abstract

The precision and safety of robotic applications rely on accurate robot models. Bayesian Neural Networks (BNNs) offer the capability to acquire intricate models and provide insights into inherent uncertainties. While recent studies have successfully employed machine learning to predict the Forward Geometric Model (FGM) of a 6-DOF (degrees of freedom) parallel manipulator, traditional methods lack predictive uncertainty estimation.

In this study, we propose a novel approach to enhance FGM prediction for a 6-RSU (Revolute-Spherical-Universal) parallel manipulator using a modified NARX-BNN (Nonlinear Autoregressive with Exogenous Inputs - Bayesian Neural Network).

The proposed NARX-BNN model benefits from a synergistic combination of the BNN structure's powerful universal approximation feature and uncertainty estimation, and the nonlinear ARX model's strong predictive capability.

The simulation and experiment results demonstrate the superiority of the proposed NARX-BNN model over traditional Bayesian shallow neural network employing the Variational inference method for this problem. At a 95 % confidence level, NARX-BNN reduces the RMSE of predicted values by up to 11 % and reduces the Average Width indicator of the prediction interval by approximately 12.7 % compared to traditional BNN.

This study underscores the potential of NARX Bayesian Neural Networks in enhancing accuracy, reducing uncertainty, and bolstering the reliability of machine learning models for robotic applications, particularly in predicting the FGM of parallel manipulators. Moreover, these advancements hold promise for improving robotic control, planning, and overall system reliability.

## 1 Introduction

Parallel manipulators play a crucial role in various industrial and research domains, requiring precise control and accurate trajectory planning. The forward geometric model (FGM), which establishes the relationship between joint angles and the resulting end-effector position and orientation, forms the basis for these tasks. However, predicting the FGM accurately for complex parallel manipulators, such as the 6-RSU configuration (where this type of manipulator employs a combination of a Revolute joint, a Spherical joint, and a Universal joint in each arm, respectively), presents significant challenges due to the intricate relationships between joint angles and end-effector pose.

In parallel mechanisms, the inverse geometric problem (IGM) is relatively straightforward and can be solved using various methods, including geometric, analytic, iterative, and screw theory approaches. However, the forward geometric analysis in parallel mechanisms is more challenging, garnering increasing attention from researchers. Numerical methods, notably the widely used Newton-Raphson method, have been employed for solving the forward geometric analysis problem. Nonetheless, these methods have limitations such as the requirement of an initial guess, convergence issues, and the potential for encountering local minimum problems. Analytical methods and sensor-based approaches offer alternative solutions, but their high cost and computational demands restrict their applicability in real-time engineering applications.

To overcome these limitations, researchers have turned to machine learning approaches, particularly Artificial Neural Networks (ANNs) [1,2], as a powerful solution for tackling models with uncertainties and highly complex nonlinearities. By training ANNs, these models can learn the system's behavior and effectively solve the forward geometric analysis problem. This application of machine learning techniques provides a promising avenue for addressing the challenges associated with uncertainties and complex nonlinearities, thereby enhancing the efficiency and accuracy of FGM in parallel mechanisms.

Recent studies have demonstrated that machine learning-based prediction of the FGM of the parallel manipulator is highly effective and practical [3]. However, traditional machine learning methods only provide prediction outcomes and lack associated predictive uncertainty. To address this limitation, various techniques such as Bayesian neural networks, ensemble methods, dropout, Monte Carlo sampling, and Gaussian processes have been developed to estimate predictive uncertainty, which is particularly useful in real-world applications where uncertainty evaluation is crucial.

The literature encompasses a wide range of parallel mechanisms that have been extensively studied, particularly in terms of their kinematic solutions [1,2]. Gao et al. [4] focused on the forward kinematic problem (FKP) of the joint variable space in the general Stewart platform mechanism. They evaluated this problem using the pseudo-arc length homotopy continuation algorithm and compared the results with those obtained using the Newton-Raphson algorithm. In a different study, Sadjadian et al. [5] proposed a combined numerical and analytical approach to solve the forward kinematics problem for a novel redundant parallel manipulator. They developed a quasi-closed form solution and incorporated various neural network architectures, including Multi-Layer Perceptron (MLP), Radial Bias Function (RBF), Probabilistic Neural Network (PNN), and Adaptive Neuro-Fuzzy Inference System (ANFIS), to enhance their approach. Furthermore, Rahmani et al. [6] presented the design and analysis of a novel redundant hybrid manipulator composed of two similar Stewart mechanisms in a serial configuration. They employed neural network training to solve the forward kinematics problem and utilized different types of neural networks, such as Multi-Layer Perceptron (MLP) and Radial Bias Function (RBF), along with various learning algorithms to approximate specific trajectories. Rahmani [7] designed and analyzed a Wavelet Neural Network (WNN) to solve the Forward Kinematics problem of a 6-RSU Co-axial Parallel Mechanism. The WNN was trained using the Final Prediction Error method, providing an effective solution for determining the mechanism's desired configurations. Chauhan et al. [8] proposed a machine learning-based solution for the forward kinematics of the Stewart platform. They utilized a multi-layer feed-forward neural network with one hidden layer, training it with different metaheuristic optimizers such as Particle Swarm Optimization (PSO), Modified Chaotic Invasive Weed Optimization (MCIWO), and Teachers' Learning-Based Optimization (TLBO) methodologies. Cursi et al. [9] introduced Bayesian Neural Networks (BNN) for estimating the highly nonlinear kinematic and dynamic models of a tendon-driven surgical robot. They leveraged the information about epistemic uncertainties through a Hierarchical Model Predictive Control (Hi-MPC) strategy, enabling effective control of the robot system. Zhu et al. [10] presented a novel hybrid algorithm based on neural networks to solve the forward kinematics problem of a 6 DOF mechanism. The proposed approach offered an effective solution for determining the desired configurations of the mechanism. Sandi et al. [11] introduced the application of a multilayer perceptron for solving the inverse kinematics problem of an industrial robotic manipulator. The results outcomes reveal a successful regression for the first five joints, while the regression for the final joint is comparatively poor due to the specific configuration of the robotic manipulator. Mohamad Reda [12] proposed the utilization of adaptive neuro-fuzzy inference systems to address the inverse kinematics problem of a 5-degree-of-freedom articulated robot. Multiple models were constructed by employing diverse deification methods, namely Subtractive Clustering (SCM), Fuzzy C-Means Clustering (FCM), and Grid Partitioning (GP), within the study.

Ho et al. [13] introduced a novel forward adaptive neural MIMO NARX model for modeling and identifying the forward kinematics of a 3-degree-of-freedom industrial robot arm system. The experimental results demonstrate that the proposed adaptive neural NARX model, trained using the Back Propagation learning algorithm, achieves exceptional performance with perfect accuracy. Kumar et al. [14] proposed a novel method based on the nonlinear autoregressive model with exogenous input (NARX) using a wavelet network for the identification of the forward kinematic model of a Stewart platform.

In the domains of computer science and robotics, considerable research efforts have been dedicated to the estimation of predictive uncertainties in machine learning (ML). In particular, the Nonlinear Autoregressive with exogenous inputs (NARX) architecture has proven effective in modeling complex systems with temporal dependencies. However, the original NARX model lacks the ability to provide uncertainty estimation, which limits its applicability in scenarios where uncertainty evaluation is essential.

In this manuscript, we propose a modified NARX Bayesian Neural Network approach (NARX-BNN) to enhance the prediction of the FGM for a 6-RSU parallel manipulator. By incorporating Bayesian principles, our modified network not only captures the intricate nonlinear relationships but also provides probabilistic predictions and uncertainty estimation. This enables a more comprehensive understanding of the predicted FGM model and enhances the reliability of control and planning algorithms.

To evaluate the performance of our proposed approach, extensive experiments Simulation and comparisons are conducted. We assess the accuracy of the FGM predictions and compare them with the traditional Bayesian shallow neural networks (BNN), highlighting the advantages and improvements achieved through our modified NARX Bayesian Neural Network. In this study, the proposed methodology is employed to address two distinct types of predictive uncertainty: aleatoric uncertainty, which arises from data noise, and epistemic uncertainty, which is attributable to limited data availability and knowledge. The ultimate goal is to assess the performance and effectiveness of the proposed NARX-BNN approach in estimating the FGM and quantifying the respective uncertainty sources.

This research makes several notable contributions to the field, including:

• Development of the proposed NARX-Bayesian neural network model specifically tailored for learning the highly nonlinear FGM of a 6-RSU parallel manipulator.
• Comparative evaluation of interval prediction performance between the proposed NARX-BNN model and the traditional one, on both Simulation and Experiment data. The assessment encompasses all coordinates of the end effector positions and orientations vector.
• Extensive analysis of the performance of the proposed model specifically for the *P*_*x*_ coordinate, utilizing two distinct datasets.

The subsequent sections of this paper are structured as follows. Section 2 provides a detailed description of the case study, which involves a Stewart platform 6-RSU parallel manipulator. The section also describes the geometric modeling of the manipulator. In Section 3, the paper discusses the proposed Bayesian neural network and the NARX neural network. Section 4 presents the simulation environment used in this study. This section provides information on the simulation setup and the parameters used in the simulations.

Section 5 presents the simulation results of the study and compares the performance of the proposed BNN models in solving the FGM problem. Section 6 presents the experiment results and provides a detailed analysis of the results and discusses the implications of the findings. Finally, in Section 7, the paper summarizes the contributions and results of the study and provides some perspectives on future research directions in this area.

## 2 Theoretical study

**Fig. 1:**
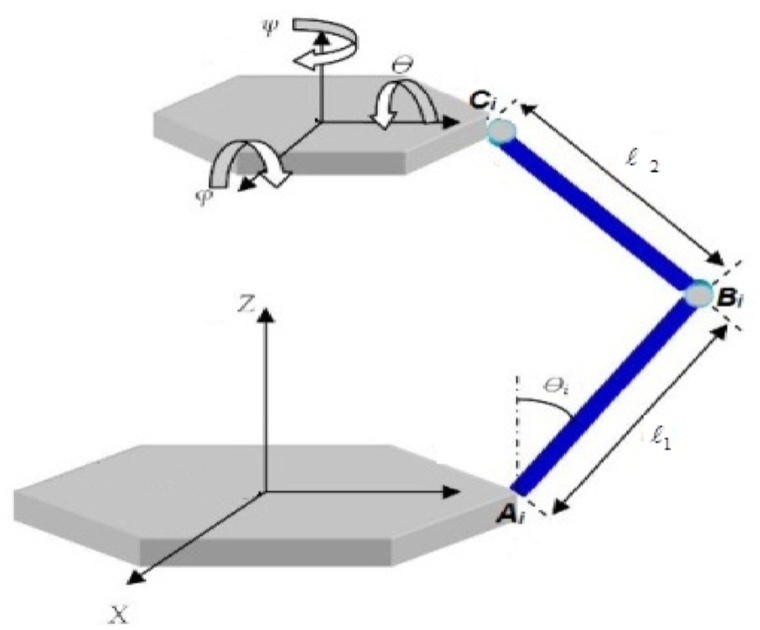
The 6-RSU mechanism.

**Fig. 2:**
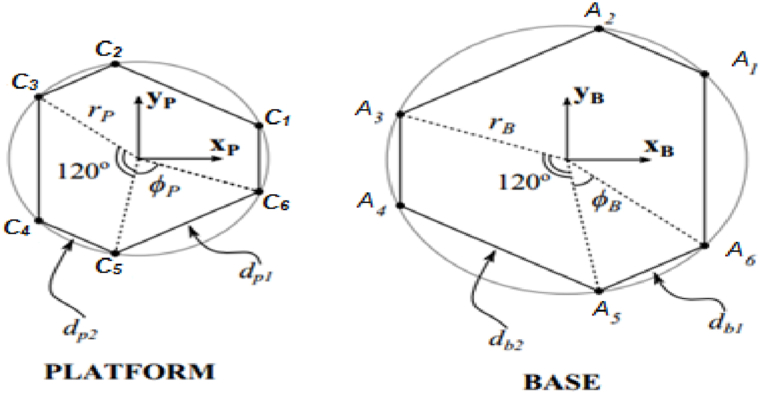
Dimensions of the two platform bases.

In the case of the 6-RSU mechanism geometry (Fig. 1), it is defined by two coplanar semi-regular hexagons: the base hexagon and the movable platform (Fig. 2). To fully describe the mechanism, at least six basic geometric parameters are required. These parameters include the lengths of the arms of the platform and the dimensions of its bases. Therefore, the vector of design parameters for the 6-RSU mechanism can be represented as follows: $P=\left[l_{1},l_{2},d_{b1},d_{b2},d_{p1},d_{p2}\right]$.

The geometric modeling of a robotic system establishes the relationship between the end-effector's location vector **X** and the joint coordinate vector **q**. Various methods and notations have been proposed for determining the geometric model, with the Denavit-Hartenberg method [15] being widely used for simple serial-structured robots. However, for more complex systems like parallel and tree structured robots, Khalil and Klein have proposed a unified description [16]. In the case of the Stewart platform, the Forward Geometric Model is defined in Equation (1) as follows:

(1) $$\begin{array}{l} X=f\left(q\right)\\ X=\left[p_{x},p_{y},p_{z},\varphi ,\theta ,\psi \right]\\ q=\left[\;\theta_{1},\theta_{2},\ldots ,\theta_{6}\;\right] \end{array}$$

Similarly, the inverse geometric model (IGM) is defined in Equation (2) as follows:

(2) $$q=f^{-1}\left(X\right)$$

In our study, the determination of the inverse geometric model is accomplished through a geometric approach, relying on a series of interrelated equations presented in Equation (3) and corresponding relationships as illustrated in Ref. [17] (see Fig. 1).

(3) $$\begin{array}{l} \theta_{i}=\mathrm{atan}\left(\sin\;\theta_{i}/\cos\;\theta_{i}\right)\\ \sin\;\theta_{i}=\frac{P_{i}\;Y_{Ci}-X_{Ci}\sqrt{\Gamma_{i}}}{X_{C_{i}}^{2}+Y_{C_{i}}^{2}}\\ \cos\;\theta_{i}=\frac{P_{i}\;X_{Ci}+Y_{Ci}\;\sqrt{\Gamma_{i}}}{X_{C_{i}}^{2}+Y_{C_{i}}^{2}}\\ :\left(\begin{array}{l} P_{i}=\frac{L_{i}^{2}+l_{1}^{2}-l_{2}^{2}}{2l_{1}},L_{i}=\left\|\vec{A_{i}C_{i}}\right\|\\ \Gamma_{i}=X_{C_{i}}^{2}+Y_{C_{i}}^{2}-{P_{i}}^{2} \end{array}\right) \end{array}$$

where *A*_*i*_*, B*_*i*_*,C*_*i*_ represent the center points of the revolute joint, spherical joint, and universal joint respectively, for each arm of the studied mechanism.

In the Forward Geometric Model, the determination of the position and orientation of the moving platform center relies on known joint-space parameters. However, due to the inherent nonlinearity of the geometric model, the mapping from known joint-space rotational angles to the pose of the moving platform becomes highly complex. To address this challenge, we propose the utilization of machine learning techniques as a powerful solution to solve the FGM problem for the 6-RSU parallel manipulator in our current work. By leveraging machine learning methods, we aim to overcome the complexities associated with the nonlinear geometric model and achieve accurate predictions of the moving platform's pose based on the given joint-space parameters.

## 3 Material and methods

### 3.1 Predictive uncertainty

Predictive uncertainty in deep learning arises from various factors, including the methodology employed for data observation, the type of model utilized, and the specific model parameters. Two distinct forms of uncertainty can be distinguished: aleatoric and epistemic uncertainty [18,19].

Aleatoric uncertainty encompasses random intrinsic variations or noise present within the data. This type of uncertainty typically arises from factors such as measurement error or noise and remains unaffected even with the collection of additional data. Aleatoric uncertainty is exacerbated in the presence of noisy data or outliers that can occur during measurements. In contrast, epistemic uncertainty accounts for the uncertainty inherent in the model itself and its associated parameters, stemming from incomplete knowledge during the model training phase. This form of uncertainty can be further subdivided into structural uncertainty, which relates to uncertainty regarding the model's structure, and uncertainty in model parameters [20].

Moreover, aleatoric uncertainty can be classified into two types: homoscedastic and heteroscedastic. Homoscedastic uncertainty refers to a constant noise function for all inputs, whereas heteroscedastic uncertainty involves variable noise functions, where the level of uncertainty varies depending on the input data.

Understanding the nature and sources of predictive uncertainty holds paramount importance in improving the accuracy and reliability of deep learning models. Addressing aleatoric and epistemic uncertainties can lead to better model performance, more robust decision-making, and increased confidence in the model's predictions.

### 3.2 Bayesian Neural Networks

Bayesian Neural Networks (BNNs) represent a specialized class of neural networks that employ the Bayesian approach to effectively incorporate and quantify uncertainty within the model [21].

In contrast to traditional neural networks that assign single point estimates to model parameters, Bayesian Neural Networks (BNNs) adopt a probabilistic framework by assigning probability distributions to these parameters [22]. This probabilistic approach enables the model to effectively capture and quantify uncertainty, thereby enhancing the robustness and reliability of the model.

BNNs are typically trained using Bayesian inference, a methodology involving the iterative update of the prior probability distribution of model parameters based on observed data. This iterative process yields a posterior probability distribution, which reflects the updated knowledge regarding the model parameters given the observed data. The posterior distribution can be leveraged to make predictions and estimate the uncertainty associated with the model outputs.

Similar to feedforward artificial neural networks (ANNs), Bayesian Neural Networks (BNNs) possess the ability to model intricate behaviors without relying on explicit mathematical or physical models. However, ANNs are susceptible to the influence of outliers within the dataset, which can result in overfitting and challenges in effectively controlling their complexity [23,24].

In contrast, BNNs address these issues by incorporating priors into the network's weights, which can help to regularize the model and prevent overfitting. By employing probability distributions to represent uncertainty in both the model parameters and predictions, BNNs provide a quantifiable measure of confidence in the model's predictions [24].

Theoretically, The BNN structure holds great potential in addressing the overfitting issue commonly encountered in deep learning neural network models, thereby enhancing prediction accuracy and generalization capabilities. Nevertheless, the utilization of BNNs also presents certain challenges [35]. Computational complexity represents a significant challenge, as it can impede efficient training and inference, leading to time-consuming processes. Additionally, selecting suitable prior distributions for the model parameters poses another challenge, as it can impact the overall performance of the model.

Despite these challenges, BNNs have become increasingly popular in recent years, as they provide a powerful tool for capturing uncertainty in deep learning models and improving their performance. Advances in computing power and optimization techniques have also helped to overcome some of the computational challenges associated with BNNs.

### 3.3 Bayesian approximation using variational inference

When training a Bayesian neural network using variational inference, the objective is to estimate the parameters of the probability distributions associated with the weights. For a given training dataset $D=\left\{x^{\left(i\right)},y^{\left(i\right)}\right\}$, assuming the data points are independent and identically distributed (*i.i.d.*), we can construct the likelihood function as presented in Equation (4):

(4) $$P\left(D\backslash w\right)=\prod_{i}P\left(y^{\left(i\right)}\backslash x^{\left(i\right)},w\right)$$

This likelihood function is a mathematical function that depends on the parameters ***w***, representing the weights of the neural network. Maximizing the likelihood function corresponds to obtaining the maximum likelihood estimation (MLE) of the parameters ***w***. In the context of training, the typical optimization objective is to minimize the negative log likelihood. In the case of a Gaussian distribution, this objective is proportional to the sum of squares error function. However, relying solely on MLE can result in significant overfitting.

By employing Bayes' theorem, multiplying the likelihood function with a prior distribution *p(w)* yields a proportionality to the posterior distribution, as expressed in Equation (5). This incorporation of the prior distribution allows for the estimation of the posterior distribution, taking into account both the observed data and the prior beliefs about the parameters ***w***.

(5) P(w\D)∝P(D\w)P(w)

Maximizing the product of the likelihood function *p(D|w)* and the prior distribution *p(w)* gives us the maximum posteriori (MAP) estimation of the parameters ***w***. The computation of the MAP estimate introduces regularization, which can effectively prevent overfitting. Both maximum likelihood estimation (MLE) and MAP estimation provide point estimates of the parameters.

However, if we had access to the full posterior distribution over the parameters, we could consider the weight uncertainty when making predictions. This is accomplished through the posterior predictive distribution [25], where the parameters have been marginalized out, as defined in Equation (6):

(6) P(y\x,D)=∫P(y\x,w)P(w\D)d(w)

In essence, this is equivalent to averaging predictions from an ensemble of neural networks, each weighted by the posterior probabilities of their respective parameters ***w***. By considering the full posterior distribution, we obtain a more comprehensive understanding of the model's predictions, incorporating the inherent uncertainty in the weights.

Unfortunately, obtaining an analytical solution for the posterior distribution *P(w|D)* in neural networks is infeasible due to its complexity. As a result, we resort to approximating the true posterior with a variational distribution q(w\θ) that has a known functional form, and we aim to estimate the parameters of this distribution. This approximation is achieved by minimizing the Kullback-Leibler (KL) divergence between the variational distribution q(w\θ) and the true posterior distribution *P(w|D).* The KL divergence quantifies the dissimilarity between two probability distributions and serves as an optimization objective to guide the estimation of the variational parameters towards a better approximation of the true posterior. The Kullback-Leibler (KL) divergence between the variational distribution q(w\θ) and the true posterior distribution *P(w|D)* is defined in Equation (7) as follows [25]:

(7) $$KL\left(q\left(w\backslash \theta \right)\|p\left(w\backslash D\right)\right)=\int q\left(w\backslash \theta \right).\log\frac{q\left(w\backslash \theta \right)}{p\left(w\backslash D\right)}\;d\left(w\right)=E_{q\left(w\backslash \theta \right)}\left[\log\frac{q\left(w\backslash \theta \right)}{p\left(w\backslash D\right)}\right]$$

By applying Bayes’ rule to *p*(*w*|*D*), we derive Equation (8) as follows:

(8) $$KL\left(q\left(w\backslash \theta \right)\|p\left(w\backslash D\right)\right)=E_{q\left(w\backslash \theta \right)}\left[\log\frac{q\left(w\backslash \theta \right)}{p\left(D\backslash w\right)p\left(w\right)}p\left(D\right)\right]=E_{q\left(w\backslash \theta \right)}\left[\log\;q\left(w\backslash \theta \right)-\log\;p\left(D\backslash w\right)-\log\;p\left(w\right)+\log\;p\left(D\right)\right]=E_{q\left(w\backslash \theta \right)}\left[\log\;q\left(w\backslash \theta \right)-\log\;p\left(D\backslash w\right)-\log\;p\left(w\right)]\right]+\log\;p\left(D\right)=KL\left(q\left(w\backslash \theta \right)\|p\left(w\right)\right)-E_{q\left(w\backslash \theta \right)}\;\log\;p\left(D\backslash w\right)+\log\;p\left(D\right)$$

using the fact that the log marginal likelihood log *p(D)* doesn't depend on w. The first two terms on Equation (8) are define as the variational free energy *F(D,θ).* Thus, we obtain Equation (9):

(9) KL(q(w\θ)‖p(w\D))=F(D,θ)+logp(D)

where the *F*(*D*,*θ*), is the corresponding optimization objective or cost function, and is derived from the Kullback-Leibler (KL) divergence, and can be expressed in Equation (10) as follows [25]:

(10) $$F\left(D,\theta \right)=KL\left(q\left(w\backslash \theta \right)\|p\left(w\right)\right)-E_{q\left(w\backslash \theta \right)}\left[\log\;p\left(D\backslash w\right)\right]$$

In this equation, the first term represents the KL divergence between the variational distribution q(w\θ) and the prior distribution *P(w).* Minimizing this term ensures that the variational distribution remains close to the prior distribution, promoting regularization and preventing overfitting. The second term represents the expected log-likelihood of the data under the variational distribution. The expectation is taken with respect to the samples drawn from the variational distribution. Maximizing this term encourages the model to fit the training data well. The overall objective is to minimize the cost function F, thereby finding the optimal parameters of the variational distribution that balance the fit to the data and the adherence to the prior.

The negative variational free energy *F*(*D*,*θ*), is also known as *evidence lower bound* L(D,θ) (ELBO), which can be expressed (as derived from Equation (9)) in Equation (11) as follows:

(11) $$\begin{array}{l} KL\left(q\left(w\backslash \theta \right)\|p\left(w\backslash D\right)\right)=-L\left(D,\theta \right)+\log\;p\left(D\right)\\ \Rightarrow L\left(D,\theta \right)=\log\;p\left(D\right)-KL\left(q\left(w\backslash \theta \right)\|p\left(w\backslash D\right)\right) \end{array}$$

Minimizing the Kullback-Leibler (KL) divergence between the posterior distribution and the variational distribution is equivalent to maximizing the evidence lower bound (ELBO), which serves as an objective function for Bayesian neural networks [26].

Indeed, variational inference offers a way to transform the Bayesian inference problem into an optimization problem. In this approach, the goal is to estimate the parameters of the variational posterior distribution, and researchers have extensively employed machine learning (ML) models, particularly neural networks, as stochastic functions to achieve this. One popular method for training the ML model in the context of variational inference is called Bayes by Backprop (BBB) [25,27]. BBB is a practical implementation of stochastic variational inference that incorporates a reparametrization trick. This trick ensures that the backpropagation algorithm can be applied effectively during the training process.

In our study, we adopt a Gaussian distribution to represent the variational posterior distribution. This distribution is parameterized by two vectors: a mean vector, denoted as ***μ***, and a standard deviation vector, denoted as ***σ***. Rather than directly parameterizing the neural network with weights, we propose to parameterize it using the mean and standard deviation vectors (***μ*** and ***σ***). This approach effectively doubles the number of parameters compared to a standard neural network, as both the mean and standard deviation vectors need to be learned. By parameterizing the neural network in this manner, we introduce additional flexibility and enable the modeling of uncertainty through the variational posterior distribution. The mean vector represents the central tendency of the distribution, while the standard deviation vector captures the level of uncertainty or variability associated with each parameter. This parameterization scheme allows us to capture the complexity of the model by incorporating uncertainty, providing a more robust and flexible framework for our study.

One of the main challenges with Bayesian neural networks lies in effectively incorporating uncertainty across the architecture, especially in deep neural networks with numerous successive layers. Accounting for uncertainty in each layer can be computationally demanding and redundant. To address this issue, hybrid Bayesian neural networks offer a practical solution. These networks strategically position only a few probabilistic layers at the end of the network, rather than throughout the entire architecture. By doing so, uncertainty estimation is focused on the final layers, where it is most critical for decision-making. This hybrid approach allows for a more efficient and scalable implementation of Bayesian neural networks. It strikes a balance between capturing uncertainty and managing computational complexity. By selectively placing probabilistic layers at the network's end, we can effectively leverage the benefits of Bayesian inference while maintaining reasonable computational costs [28].

### 3.4 NARX neural network

The Nonlinear Autoregressive with Exogenous Inputs (NARX) model is a recurrent dynamic network comprising feedback connections that encompass multiple network layers. The NARX model is derived from the linear Autoregressive with Exogenous Inputs (ARX) model, which is a widely used approach for time-series modeling.

The NARX model integrates the principles of autoregressive models and exogenous inputs to capture the dynamic behavior of a system. Autoregressive models describe the present output of a system as a function of its previous outputs, while exogenous inputs are additional variables that can influence the system's behavior.

The NARX model is particularly useful in cases where the system being modeled exhibits nonlinear behavior and is influenced by external factors. By incorporating both autoregressive terms and exogenous inputs, it can capture complex dynamics and make predictions based on historical data and current inputs.

The NARX model is characterized by Equation (12), which is expressed as follows:

(12) $$y\left(t\right)=f\left(\;y\left(t-1\right),y\left(t-2\right),...,y\left(t-n_{y}\right),u\left(t-1\right),u\left(t-2\right),...,u\left(t-n_{u}\right)\;\right)$$

This equation represents the regression of the future value of the dependent output signal y(t) upon past values of both the output signal and an independent (exogenous) input signal.

**Fig. 3:**
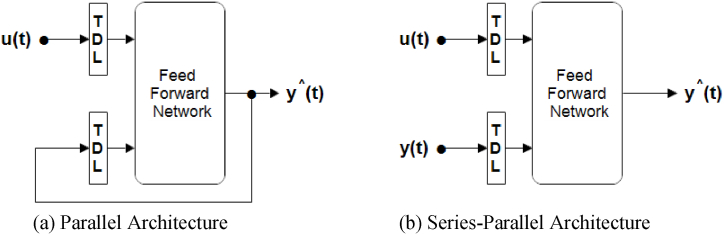
The configuration of the NARX model.

Regarding the configuration, the standard NARX Parallel architecture involves connecting the output of the NARX network back to the input of the feedforward neural network, as shown in Fig. 3(a). However, during the network training phase, the availability of the true output allows for the adoption of a series-parallel architecture [29]. In this architecture, the true output is utilized instead of the estimated output within the feedback loop, as depicted in Fig. 3(b). This approach provides two significant advantages. Firstly, it leads to a more accurate input provided to the feedforward network. Secondly, the resulting network exhibits a purely feedforward structure, enabling the utilization of static backpropagation for training purposes.

The NARX network encompasses a wide range of applications. It serves as a predictive tool, enabling the estimation of the subsequent value of the input signal. Furthermore, it finds utility in nonlinear filtering, where the desired output corresponds to a denoised rendition of the input signal. Another significant application of the NARX network lies in the modeling of nonlinear dynamic systems.

### 3.5 Model evaluation

The Prediction Interval Coverage Probability (PICP) and Prediction Interval Average Width (PIAW) are widely used metrics for evaluating interval prediction models [30,31].

The Prediction Interval Coverage Probability (PICP) assesses the ability of a model to accurately capture the true values within the generated prediction intervals. It is defined as the proportion of true values that lie within the prediction intervals. The formula for calculating PICP is given in Equation (13) as follows:

(13) $$PICP=\frac{1}{N}\sum_{n=1}^{N}S_{n}$$

where *N* represents the total number of FGM points to be predicted, and *S*_*n*_ is a Boolean function. The function *S*_*n*_ takes the value of 1 when both the upper and lower boundaries of the FGM prediction interval encompass the true value. Conversely, when the true value falls outside the prediction interval, the function *S*_*n*_ takes the value of 0.

On the other hand, the Prediction Interval Average Width (PIAW) is a metric used to quantify the average width of the prediction intervals generated by the model. It is calculated as the average difference between the upper and lower bounds of the prediction intervals. The formula for calculating PIAW is given in Equation (14) as follows:

(14) $$PIAW=\frac{1}{N}\sum_{n=1}^{N}\left(P_{up}-P_{down}\right)$$

where the terms *P*_*up*_ and *P*_*down*_ represent the upper and lower bound values of the prediction interval, respectively.

Indeed, in evaluating interval forecast models, a desirable model would exhibit a high Prediction Interval Coverage Probability (PICP) and a low Prediction Interval Average Width (PIAW). Thus, a good interval forecast model would demonstrate both a high PICP and a low PIAW, signifying accurate and precise prediction intervals.

The root mean square error (RMSE) and mean absolute error (MAE) are commonly used deterministic evaluation metrics to assess the accuracy of model predictions [32,33]. The mean absolute error (MAE) is defined in Equation (15) as follows:

(15) $$MAE=\frac{1}{N}\sum_{n=1}^{N}\left|P_{tv}-P_{pv}\right|$$

where *P*_*tv*_ represents the true value and *P*_*pv*_ represents the corresponding predicted value. The root mean square error (RMSE) is defined in Equation (16) as follows:

(16) $$RMSE=\sqrt{\frac{1}{N}\sum_{n=1}^{N}{\left(P_{tv}-P_{pv}\right)}^{2}}$$

Both the MAE and RMSE are widely used metrics to evaluate the accuracy of model predictions, with lower values indicating better predictive performance.

## 4 Simulation environment

**Fig. 4:**
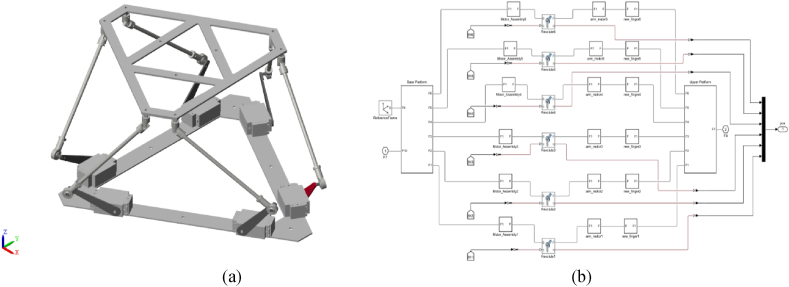
The simulated diagram of the considered 6-RSU mechanism.

For the simulated environment, Sim-Scape MATLAB was employed. Similarly to the real case scenario, an approximated geometric model of the considered 6-RSU mechanism was built. Fig. 4(a) illustrates the simulated 6-RSU parallel manipulator, while Fig. 4(b) shows the corresponding diagram of the considered mechanism. The geometrical specifications of the considered 6-RSU mechanism-Zamanov type are summarized in Table 1.

**Table 1:** Geometrical specifications of 6-RSU mechanism.

parameter	$d_{b1}$	$d_{b2}$	$d_{p1}$	$d_{p2}$	$l_{1}$	$l_{2}$
Value (cm)	**26**	**20**	**30**	**6**	**11**	25.5

In order to create an accurate forward geometric model of the 6-RSU mechanism, we collected enough data to represent the workspace of the mechanism. This involved regularly moving the arms and recording the values of joint angles ($q=\left[\theta_{1},\theta_{2},\theta_{3},\theta_{4},\theta_{5},\theta_{6}\right]$) as well as the positions and orientations of the moving platform ($X=\left[p_{x},p_{y},p_{z},\varphi ,\theta ,\psi \right]$) in the simulated model.

The whole workspace of the analyzed mechanism can be represented using a Cartesian cube. This cube is defined by specific values for the ranges of coordinates, which are as follows:

$$\begin{array}{l} p_{x}:\left[-5,5\right]cm,p_{y}:\left[-5,5\right]cm,p_{z}:\left[18,28\right]cm\\ \varphi :\left[-2,2\right]\deg,\theta :\left[-2,2\right]\deg,\psi :\left[-2,2\right]\deg \end{array}$$

Additionally, the dataset used for this study was collected from the mechanism's workspace and consisted of 20,000 data points. Subsequently, the collected data points were partitioned into three distinct subsets: a training set comprising 70 % of the data, a validation set consisting of 15 %, and a testing set also encompassing 15 %. The training set served as the basis for training the model to capture the underlying patterns in the data samples. The validation set played a crucial role in monitoring the learning process of the network and assessing the quality of the trained model. Finally, the testing set was utilized to evaluate and assess the predictive performance of the developed model.

### 4.1 Network architecture

In this paper, to build the proposed NARX-BNN model, Tensorflow Probability framework was used. The parameters used in the coding and simulation environment are summarized in Table 2.

**Table 2:** The simulation parameters.

*parameter*	Time step	Optimizer	Learning rate	Normalizer	Regularizer	Loss function	Prior distribution	Earley Stopping
***Value***	***0.1 (sec)***	***Adam***	***0.001***	***min-max (-1,1)***	***L2*** ***0.0001***	**negative log-likelihood**	**Normal (0,1)**	**15**

**Fig. 5:**
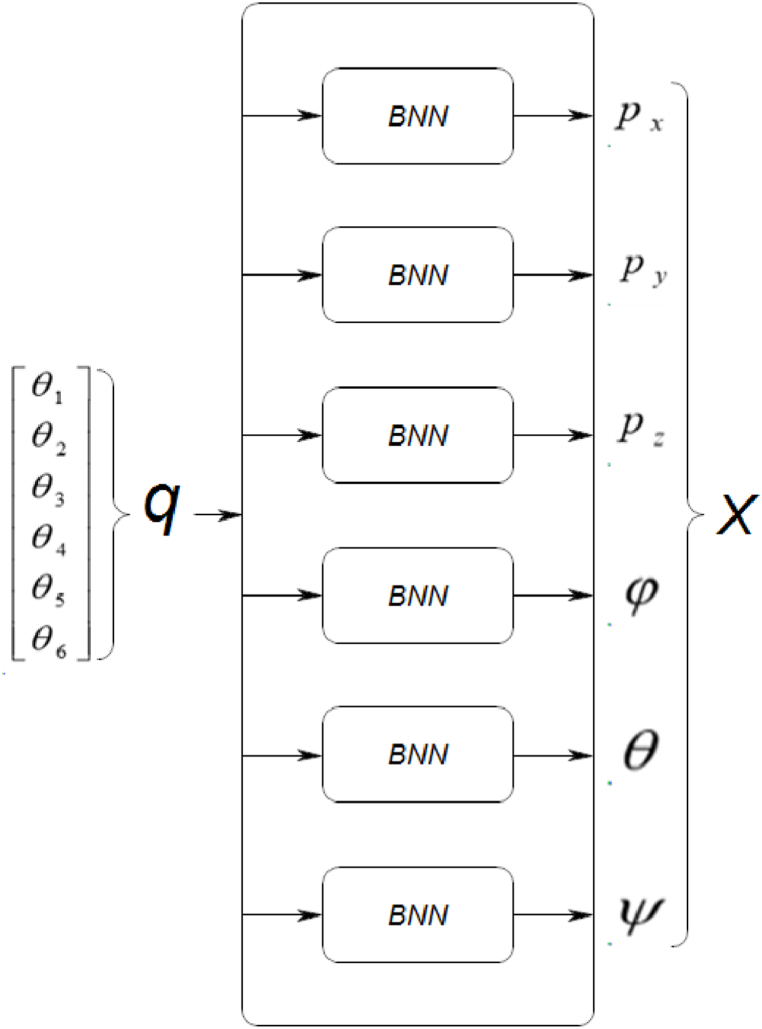
The proposed network architecture of the Forward geometric model of the 6-RSU mechanism.

To create the forward geometric model, we utilized six separate networks (as depicted in Fig. 5), with each one dedicated to a specific coordinate in the Cartesian position and orientation vector **X** of the 6-RSU mechanism's end effector. The input for each network consisted of a 6-dimensional vector representing the joint angle values **q**.

### 4.2 NARX-BNN architecture

The NARX network has been modified to enhance its performance specifically for predicting the behavior of the 6-RSU parallel manipulator in the FGM model. Our study introduces changes to the network structure, referred to as the Correctional BNN architecture.

**Fig. 6:**
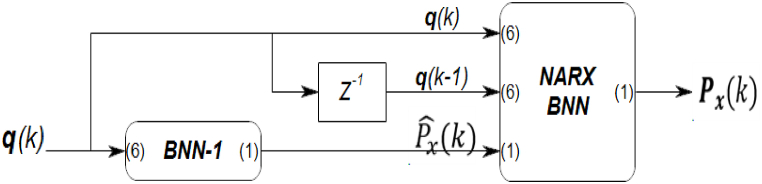
The structure of the proposed Correctional BNN model.

The series-parallel architecture was employed for this modification. Within this architecture, the pre-estimated output, generated using a traditional Bayesian neural network (BNN-1), is utilized in the feedback loop instead of the true output, as illustrated in Fig. 6.

These modifications aim to overcome the challenges and limitations encountered with the original NARX model, including the improvement of prediction accuracy, as well as the handling of noisy and incomplete input data.

In this study, the Random search algorithm was utilized to determine the optimal configuration of hidden layers and the number of neurons within those layers for the two proposed models, BNN-1 and NARX-BNN. Furthermore, we investigated the impact of different activation functions, including *Relu, Sigmoid*, and *Swish*, on the performance of these models. The selection of model parameters was guided by the objective of minimizing the loss function on the validation dataset, thereby ensuring the attainment of optimal model performance.

**Fig. 7:**
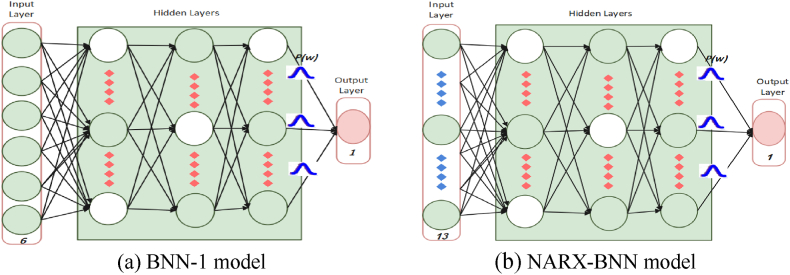
The structure of the proposed BNN models.

The proposed NARX-BNN model architecture is depicted in Fig. 7(b), comprises an input layer with thirteen neurons, three hidden layers with 16 dense neurons, 10 dense neurons, and 6 dense variational neurons (respectively), and the *Swish* activation function [34], which offers the advantages of both *relu* and *sigmoid* activation functions while ensuring continuity in the derivatives. The model outputs consist of the predictive posterior distributions of the FGM, as well as their corresponding aleatoric and epistemic uncertainties.

Similarly, the BNN-1 model architecture, shown in Fig. 7(a), comprises an input layer with six neurons, three hidden layers with 16 dense neurons, 10 dense neurons, and 4 dense variational neurons(respectively). The *Swish* activation function is also employed. The model outputs include predictive posterior distributions of the FGM.

To train the network, we utilized the Bayesian backpropagation algorithm, with the negative log-likelihood as a loss function. During the Bayesian backpropagation process, we employed the *adam* optimizer. The training process commenced with the BNN-1 model, which was trained first. Subsequently, the trained BNN-1 model was utilized to train the NARX-BNN model.

To prevent overfitting, we applied the early stop strategy to both models (BNN-1 and NARX-BNN). Additionally, considering the dataset's size, the networks were trained using a batch size of 64 units and a maximum limit of 200 epochs.

## 5 Simulation results

In this section, simulation results are presented and discussed to compare the effectiveness of the two proposed Bayesian neural networks (BNN-1 & NARX-BNN) in solving the FGM problem. The performance of the BNN models was evaluated using two different datasets. The first dataset (*dataset-1*) was collected from the whole workspace of 6-RSU simulator (as mentioned before) which was used to evaluate the FGM prediction performance of the proposed BNN models. Meanwhile, the second one (*dataset-2*) was collected from the whole workspace except some regions within it, and this dataset was used to evaluate the epistemic uncertainty prediction performance. White variable noise (heteroscedastic uncertainty) is then added to all the measurements to evaluate the aleatoric uncertainties prediction performance.

Additionally, to evaluate the generalization performance of the proposed BNN models, we applied a *Testing path* within the workspace of the studied 6-RSU mechanism. This path followed a spiral trajectory and was defined by the following equations:

⁃ *X = 4* sin*(t)/cm/*
⁃ *Y = 4* cos*(t)/cm/*
⁃ *Z = 19* + *8t/2pi/cm/*
⁃ *Phi* = *(-2)* cos*(t)/deg/*
⁃ *Theta* = *(-2)* sin*(t)/deg/*
⁃ *Psi* = *(-2)* cos*(t)/deg/*

where *t* varies between 0 and 2*pi*.

### 5.1 The interval prediction performance of the P_x_ coordinate on dataset-1

**Fig. 8:**
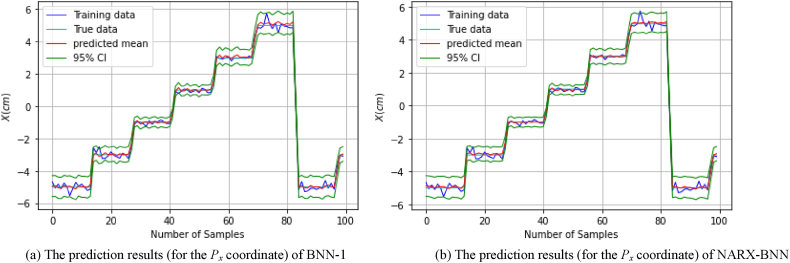
The prediction results (for the *P*_*x*_ coordinate) of two BNN models on *Testing set-dataset-1*.

Fig. 8(a) shows the prediction results of the testing set (of *dataset-1*), and the total uncertainty therein for BNN-1, while Fig. 8(b) shows the prediction results and uncertainty therein for the NARX-BNN. The evaluation results of the interval and the predicted value of the BNNs models are shown in Table 3.

**Table 3:** Evaluation of FGM (for the *P*_*x*_ coordinate) prediction results of two BNN models on *Testing set-dataset-1*.

Dataset-1 *(Testing set)*	PICP	PIAW *(mm)*	MAE *(mm)*	RMSE *(mm)*
*BNN-1*	**1.0**	9.4	0.48	0.60
*NARX-BNN*	**1.0**	**8.9**	**0.33**	**0.43**

The average training time for the BNN-1 model was about 45.9 s on the first dataset (*dataset-1)*. Meanwhile the average training time for the NARX-BNN model was 48.8 s.

**Fig. 9:**
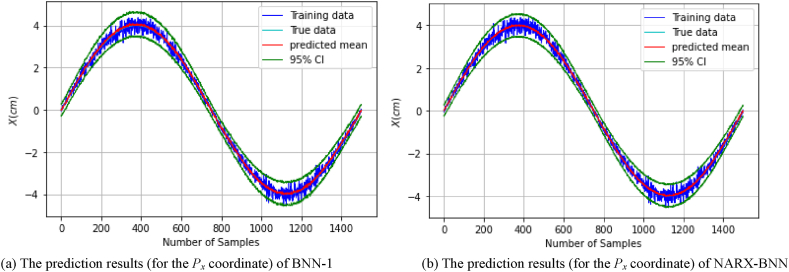
The prediction results (for the *P*_*x*_ coordinate) of two BNN models on *Testing path-dataset-1*.

Furthermore, in order to assess the generalization performance of the proposed BNN models in the FGM problem, the testing path of the *P*_*x*_ coordinate was employed and tested on the trained models. The prediction results of the testing path and the total uncertainty for the BNN-1 and the NARX-BNN models are presented in Fig. 9. Specifically, Fig. 9(a) displays the prediction results for BNN-1, while Fig. 9(b) shows the prediction results for the NARX-BNN.

Fig. 8, Fig. 9 visually illustrate the effectiveness of the proposed BNN models, in predicting the FGM for the *P*_*x*_ coordinate. The 95 % confidence level prediction intervals depicted in the figures successfully encompass the true value, highlighting the accuracy and reliability of the BNN models in making precise predictions. Moreover, the confidence interval width values of the two BNN models are incrementally variable and compatible with the heteroscedastic noise added to the measurements, demonstrating the effectiveness of the aleatoric uncertainty prediction performance.

Fig. 9 illustrates a notable aleatoric uncertainty observed in both BNN models within the region surrounding *P*_*x*_ = ±4 cm. This uncertainty is attributed to the substantial noise levels inherent in the training data at those specific locations.

Furthermore, the interval evaluation indicators (PICP, PIAW), demonstrate that the coverage values for the prediction intervals generated by both BNN-1 and the NARX-BNN models are similar. However, it is noteworthy that the prediction intervals produced by the NARX-BNN have a narrower width compared to BNN-1 (as shown in Table 3, Table 4).

**Table 4:** Evaluation of FGM (for the *P*_*x*_ coordinate) prediction results of two BNN models on *Testing path-dataset-1*.

*Testing path*	PICP	PIAW *(mm)*	MAE *(mm)*	RMSE *(mm)*
*BNN-1*	**1.0**	8.7	0.36	0.44
*NARX-BNN*	**1.0**	**8.5**	**0.21**	**0.26**

In addition, the deterministic predictive evaluation indicators (MAE, RMSE), suggest that NARX-BNN outperforms BNN-1 in terms of prediction accuracy and generalization capabilities (as indicated in Table 4). These findings indicate that the proposed NARX-BNN provides more accurate predictions and demonstrates better generalization performance compared to BNN-1.

### 5.2 The interval prediction performance of the P_x_ coordinate on dataset-2

*dataset-2* was collected from the whole workspace except the regions of *P*_*x*_ coordinate between]-3,3[cm. These data were stacked together into 13350 data points, which were then split into a training set (70 %), validation set (15 %), and a testing set (15 %) (as mentioned before).

**Fig. 10:**
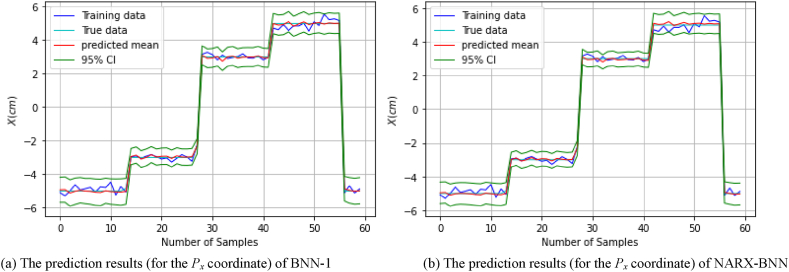
The prediction results (for the *P*_*x*_ coordinate) of two BNN models on *Testing set-dataset-2*.

Fig. 10(a) shows the prediction results of the testing set (of *dataset-2*), and uncertainty therein for BNN-1, while Fig. 10(b) shows the prediction results, and uncertainty therein for the NARX-BNN. Table 5 presents the evaluation results for the prediction interval and predicted values of the BNNs models.

**Table 5:** Evaluation of FGM (for the *P*_*x*_ coordinate) prediction results of two BNN models on *Testing set-dataset-2*.

Dataset-2 *(Testing set)*	PICP	PIAW *(mm)*	MAE *(mm)*	RMSE *(mm)*
*BNN-1*	**1.0**	12.1	0.79	0.99
*NARX-BNN*	**1.0**	**10.3**	**0.40**	**0.52**

**Fig. 11:**
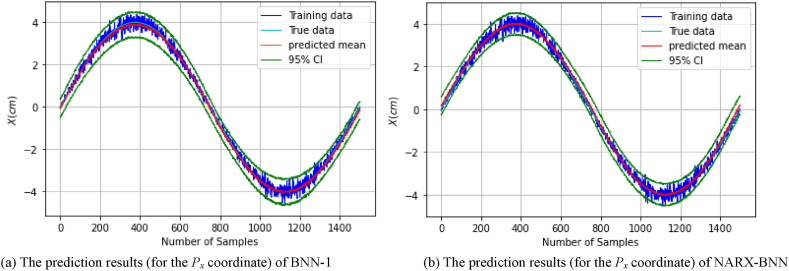
The prediction results (for the *P*_*x*_ coordinate) of two BNN models on *Testing path-dataset-2*.

Furthermore, in order to assess the generalization performance in this case, the testing path of the *P*_*x*_ coordinate was also employed and tested on the trained models. The prediction results of the testing path and the total uncertainty for the BNN-1 and the NARX-BNN models are presented in Fig. 11. Specifically, Fig. 11(a) shows the prediction results for BNN-1, while Fig. 11(b) shows the prediction results for the NARX-BNN.

Fig. 10, Fig. 11 provide visual evidence supporting the effectiveness of the proposed BNNs models in accurately predicting the FGM for Case-2. The 95 % confidence level prediction interval, depicted in the figures, exhibits complete coverage of the true value. This outcome validates the robustness and reliability of the BNN models in accurately estimating the FGM for Case-2, as the prediction intervals effectively account for the inherent uncertainty and variability in the data.

Furthermore, Fig. 11 demonstrates the occurrence of significant epistemic uncertainty in both BNN models within the *P*_*x*_ = ]-3,3[ cm region. This uncertainty arises from the limited availability of training data, which hinders the accurate determination of model parameters.

Specifically, the lack of training data in the regions between −3 cm and 3 cm in dataset-2 contributes to this high epistemic uncertainty. This reflects the effectiveness of the epistemic uncertainty prediction performance, particularly in the case of the NARX-BNN model. Meanwhile, in a similar manner to the previous case (Fig. 9), within the Px = ]-3,3[ cm region, we observe a narrower epistemic uncertainty. This is due to the training data distribution (*dataset-1*) encompassing the entirety of the testing path region.

Based on the deterministic predictive evaluation indicators (MAE, RMSE) in Table 6, the deviation between the predicted value and the true value of the interval prediction in the case of dataset-2 is greater than in the case of dataset-1 (Table 4). Additionally, the results indicate that the NARX-BNN model has better interval prediction performance of *P*_*x*_ on both datasets. The NARX-BNN provides more accurate predictions and better generalization.

**Table 6:** Evaluation of FGM (for the *P*_*x*_ coordinate) prediction results of two BNN models on *Testing path-dataset-2*.

*Testing path*	PICP	PIAW *(mm)*	MAE *(mm)*	RMSE *(mm)*
*BNN-1*	**1.0**	10.2	0.66	0.79
*NARX-BNN*	**1.0**	**8.9**	**0.55**	**0.78**

### 5.3 The interval prediction performance of the remaining FGM coordinates

In this case, the 95 % confidence interval prediction results of the remaining coordinates of the end effector positions and orientations vector $\left(p_{y},p_{z},\varphi ,\theta ,\psi \right)$ were examined.

**Fig. 12:**
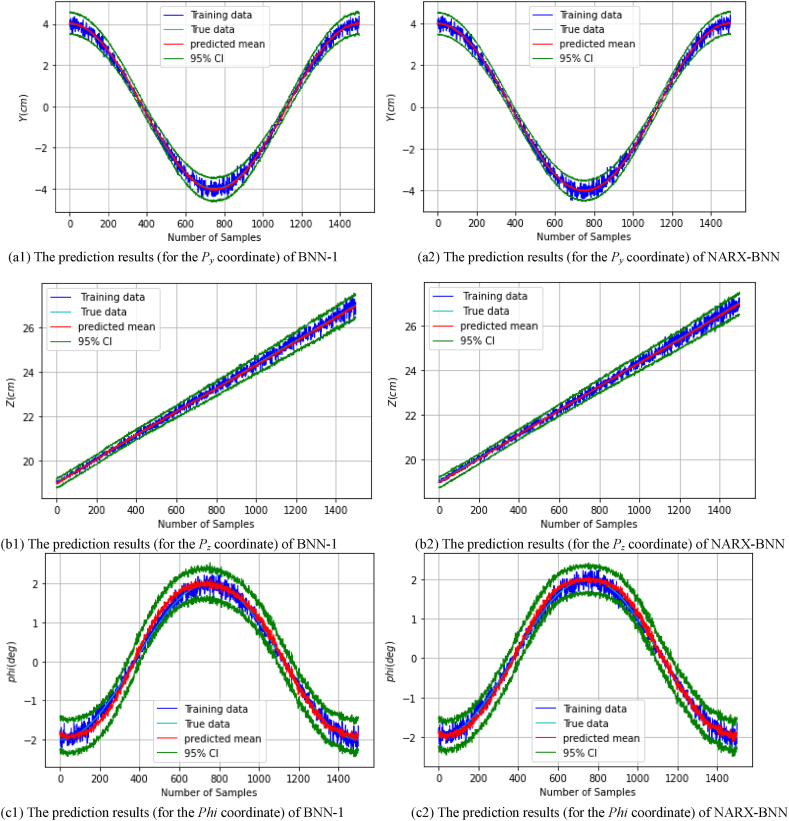

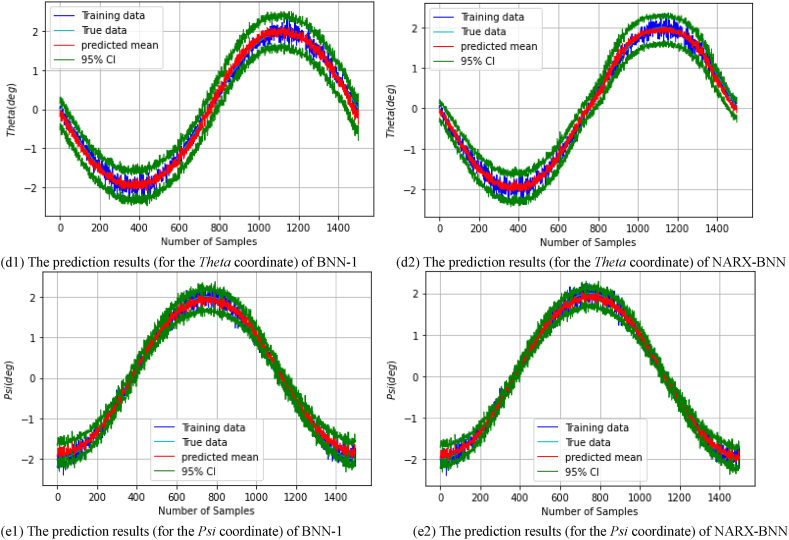
The prediction results (for the rest coordinates) of two BNN models on **Simulation***Testing paths-dataset-1*.

After performing the simulation and training each model on the *dataset-1*, the FGM solution and the total uncertainty therein can be estimated. Fig. 12 show the prediction results of the testing paths of the rest coordinates, and the total uncertainty therein for BNN-1 and the NARX-BNN. Specifically, Fig. 12(a1) shows the prediction results of *P*_*y*_ coordinate for BNN-1, while Fig. 12(a2) shows the prediction results of *P*_*y*_ coordinate for the NARX-BNN. Similarly, Fig. 12(b1) shows the prediction results of *P*_*z*_ coordinate for BNN-1, whereas Fig. 12(b2) shows the prediction results of *P*_*z*_ coordinate for the NARX-BNN.

Fig. 12(c1) shows the prediction results of *Phi* coordinate for BNN-1, while Fig. 12(c2) shows the prediction results of *Phi* coordinate for the NARX-BNN. Additionally, Fig. 12(d1) shows the prediction results of *Theta* coordinate for BNN-1, whereas Fig. 12(d2) shows the prediction results of *Theta* coordinate for the NARX-BNN. Finally, Fig. 12(e1) shows the prediction results of *Psi* coordinate for BNN-1, while Fig. 12(e2) shows the prediction results of *Psi* coordinate for the NARX-BNN.

The evaluation results of the interval and the predicted values of the all BNNs models are shown in Table 7.

**Table 7:** Evaluation of FGM prediction results (for the rest coordinates) of two BNN models on **Simulation***Testing paths-dataset-1*.

*FGM* coordinates	*Testing path*	PICP	PIAW (mm)	MAE (mm)	RMSE (mm)
*P*_*y*_	*BNN-1*	**1.0**	8.3	0.36	0.42
	*NARX-BNN*	**1.0**	**7.7**	**0.24**	**0.299**
*P*_*z*_	*BNN-1*	**1.0**	6.5	0.36	0.44
	*NARX-BNN*	**1.0**	**6.1**	**0.29**	**0.36**
		**PICP**	**PIAW (deg)**	**MAE (deg)**	**RMSE (deg)**
Phi (φ)	*BNN-1*	**1.0**	0.77	0.10	0.13
	*NARX-BNN*	**1.0**	**0.72**	**0.08**	**0.10**
Theta (θ)	*BNN-1*	**0.99**	0.76	0.085	0.11
	*NARX-BNN*	**0.99**	**0.61**	**0.06**	**0.08**
Psi (ψ)	*BNN-1*	**0.95**	0.44	0.086	0.11
	*NARX-BNN*	**0.95**	**0.38**	**0.06**	**0.08**

The 95 % confidence level prediction interval, depicted in Fig. 12, demonstrates complete coverage of the true value. This observation validates the robustness and reliability of the BNN models in accurately estimating the FGM for all coordinates, Additionally, the interval width values of the two BNN models are incrementally variable and compatible with the heteroscedastic noise added to the measurements, demonstrating the effectiveness of the aleatoric uncertainty prediction performance, which is similar to the previous case.

Table 8 presents the average values of transition and rotation indicators of the two BNN models. The results indicate that at a 95 % confidence level, the average RMSE of the predicted transition value ($p_{x},p_{y},p_{z}$) can be reduced by up to 28 % from BNN-1 to the proposed NARX-BNN, while the average RMSE of the predicted rotation value (φ,θ,ψ) can be reduced by up to 25 %. Consequently, the average RMSE of all predicted value can be reduced by approximately 27 %.

**Table 8:** Average values of transition and rotation indicators of the two BNN models based on **Simulation** results.

**Average values of indicators**		PICP	PIAW	MAE	RMSE
*Transition****(mm)***	*BNN-1*	**1.0**	7.8	0.36	0.43
	*NARX-BNN*	**1.0**	**7.4**	**0.25**	**0.31**
*Rotation****(deg)***	*BNN-1*	**0.98**	0.66	0.09	0.12
	*NARX-BNN*	**0.98**	**0.57**	**0.07**	**0.09**

Furthermore, Table 8 also indicates that the NARX-BNN produces a narrower interval width, with an average PIAW (prediction interval Average Width) for all coordinates can be enhanced by approximately 5 %, from BNN-1 to the NARX-BNN.

Therefore, the study concludes that the NARX-BNN provides more accurate predictions and more reliable uncertainty estimates than BNN-1 in predicting the forward geometric model of the 6-RSU parallel manipulator.

## 6 Experiment results and discussion

**Fig. 13:**
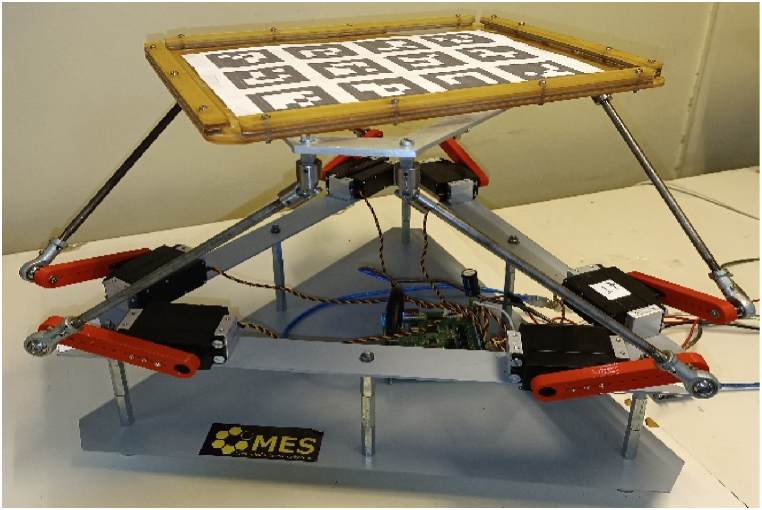
The Real 6-RSU parallel manipulator.

This section presents and discusses experimental results that validate the effectiveness of the proposed NARX-BNN model in solving the FGM problem. The experiments were conducted on a real 6-RSU parallel platform (Fig. 13) with the parameters summarized in Table 1. The platform is actuated by geared Hitec-HS-805MG closed-loop servo motors with a holding torque of 2.4 Nm.

To measure the position and orientation of the moving platform, an ArUco markers was utilized in conjunction with an OpenCV Python program and a camera system with a resolution of 720x480 pixels and a frame rate of 30 frames per second (fps). This experimental setup enabled the acquisition of ground truth data for evaluating the performance of the BNN-based FGM solution.

**Fig. 14:**
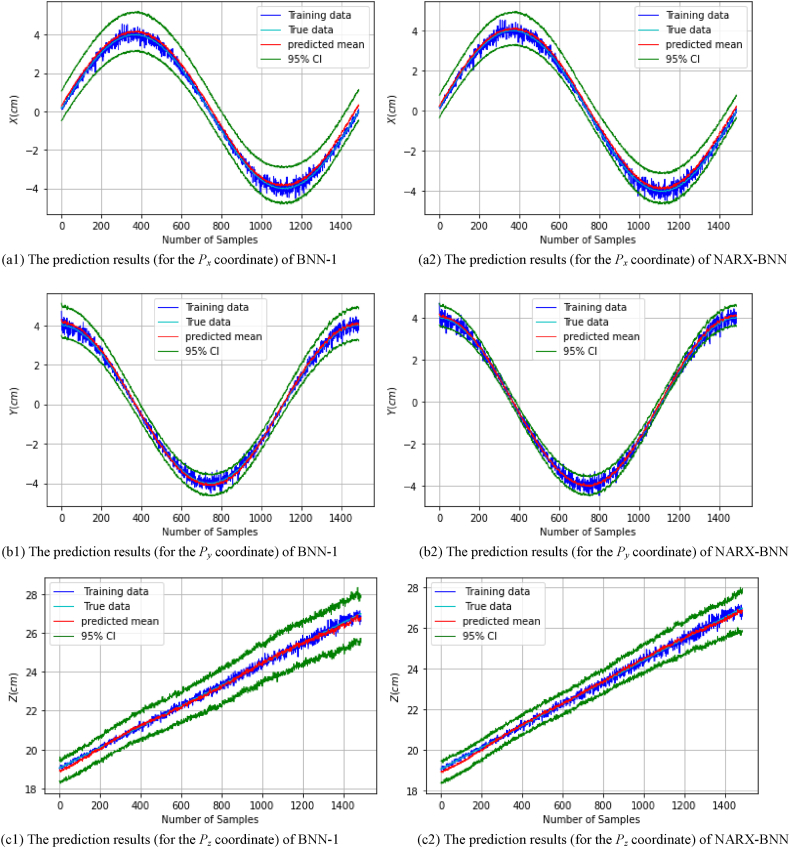

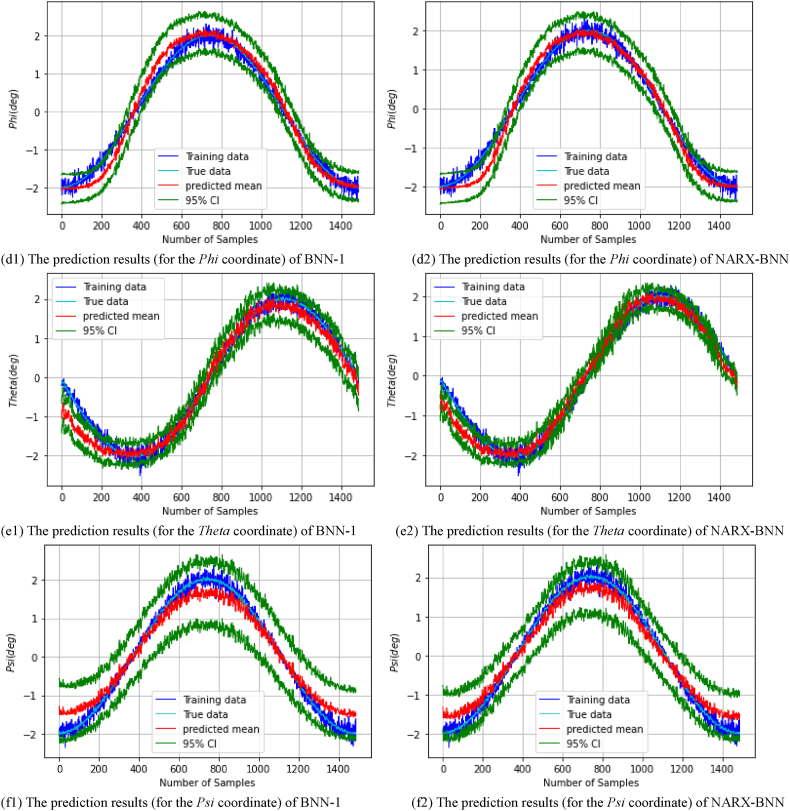
The prediction results for the all **FGM** coordinates of two BNN models on **Experiment***Testing paths-Real dataset-1*.

After performing the experiments and training each model on the real *dataset-1*, the FGM solution and the total uncertainty therein can be estimated. Fig. 14 show the prediction results of the testing paths of the all coordinates $\left(p_{x},p_{y},p_{z},\varphi ,\theta ,\psi \right)$, and the total uncertainty therein for BNN-1 and NARX-BNN models. Specifically, Fig. 14(a1) shows the prediction results of *P*_*x*_ coordinate for BNN-1, while Fig. 14(a2) shows the prediction results of *P*_*x*_ coordinate for the NARX-BNN. Similarly, Fig. 14(b1) shows the prediction results of *P*_*y*_ coordinate for BNN-1, whereas Fig. 14(b2) shows the prediction results of *P*_*y*_ coordinate for the NARX-BNN. Additionally, Fig. 14(c1) shows the prediction results of *P*_*z*_ coordinate for BNN-1, whereas Fig. 14(c2) shows the prediction results of *P*_*z*_ coordinate for the NARX-BNN.

Fig. 14(d1) shows the prediction results of *Phi* coordinate for BNN-1, while Fig. 14(d2) shows the prediction results of *Phi* coordinate for the NARX-BNN. Additionally, Fig. 14(e1) shows the prediction results of *Theta* coordinate for BNN-1, whereas Fig. 14(e2) shows the prediction results of *Theta* coordinate for the NARX-BNN. Finally, Fig. 14(f1) shows the prediction results of *Psi* coordinate for BNN-1, while Fig. 14(f2) shows the prediction results of *Psi* coordinate for the NARX-BNN.

The evaluation results of the interval and the predicted values of the all BNNs models are shown in Table 9.

**Table 9:** Evaluation of FGM prediction results (for the all coordinates) of two BNN models on Experiment *Testing paths-Real dataset-1*.

*FGM* coordinates	*Testing path*	PICP	PIAW (mm)	MAE (mm)	RMSE (mm)
*P*_*x*_	*BNN-1*	**1.0**	17.4	1.85	1.94
	*NARX-BNN*	**1.0**	**13.5**	**1.59**	**1.69**
*P*_*y*_	*BNN-1*	**1.0**	10.9	0.77	0.90
	*NARX-BNN*	**1.0**	**7.04**	**0.53**	**0.67**
*P*_*z*_	*BNN-1*	**1.0**	17.5	0.83	1.03
	*NARX-BNN*	**1.0**	**12.5**	**0.77**	**0.96**
		**PICP**	**PIAW (deg)**	**MAE (deg)**	**RMSE (deg)**
Phi (φ)	*BNN-1*	0.91	0.84	0.17	0.22
	*NARX-BNN*	**0.92**	**0.83**	**0.16**	**0.21**
Theta (θ)	*BNN-1*	0.85	0.69	0.18	0.25
	*NARX-BNN*	**0.87**	**0.49**	**0.16**	**0.22**
Psi (ψ)	*BNN-1*	**1.0**	1.42	0.24	0.29
	*NARX-BNN*	**1.0**	**1.17**	**0.22**	**0.26**

The experimental data is observed to be noisy and less accurate compared to the simulation data, likely due to the performance limitations of the servo motor and the measurement capabilities of the camera system employed. Even though the experimental results demonstrate the robustness and reliability of the proposed NARX-BNN model in estimating the FGM of the 6-RSU parallel platform.

The 95 % confidence level prediction intervals partly encompass the true values, validating the models' performance, however, there is lower accuracy in the deterministic predictive evaluation indicators (as shown in Table 9) compared to the simulation results. Furthermore, the interval width values of the BNN models are compatible with the heteroscedastic noise added to the measurements, showcasing the effectiveness of the aleatoric uncertainty prediction, similar to the simulation findings.

Quantitative analysis of the experiments results presented in Table 10 highlights the performance improvements achieved by the NARX-BNN model. Compared to BNN-1, the average RMSE of the predicted transition values is reduced by up to 13.9 % in NARX-BNN, while the average RMSE of the predicted rotation values is reduced by up to 8 %. Overall, the average RMSE of all predicted values is reduced by approximately 11 %.

**Table 10:** Average values of transition and rotation indicators of the two BNN models based on Experiment results.

**Average values of indicators**		PICP	PIAW	MAE	RMSE
*Transition****(mm)***	*BNN-1*	**1.0**	15.3	1.15	1.29
	*NARX-BNN*	**1.0**	**11.0**	**0.96**	**1.11**
*Rotation****(deg)***	*BNN-1*	0.92	0.98	0.20	0.25
	*NARX-BNN*	**0.93**	**0.83**	**0.18**	**0.23**

Additionally, Table 10 also indicates that NARX-BNN produces a narrower interval width, with an average reduction of approximately 12.7 % for all coordinates compared to BNN-1, similar to the simulation findings.

Therefore, the study concludes that NARX-BNN provides more accurate predictions and more reliable uncertainty estimates than BNN-1 in predicting the forward geometric model of the 6-RSU parallel manipulator.

Finally, these experimental findings show that the NARX Bayesian approach can significantly improve the accuracy of FGM prediction while reducing the associated uncertainty, which is crucial for precise and safe control of manipulator movements.

Overall, the proposed approach offers several potential benefits. Accurate FGM model prediction is crucial for precise robot control, motion planning, and trajectory optimization. By leveraging the power of neural networks and Bayesian inference, the modified NARX-BNN model can provide more reliable and robust predictions, enabling better control and planning of the 6-RSU parallel manipulator.

The results of this study contribute to the growing body of knowledge in neural network-based modeling techniques for robotic systems, paving the way for further advancements in this field.

## 7 Conclusions

This study presents a novel approach to enhance the prediction of the forward geometric model (FGM) for a 6-RSU parallel manipulator and predict its uncertainties, through the application of a modified NARX Bayesian Neural Network. Specifically, the NARX architecture, known for its capability to capture nonlinear dynamics, is modified to incorporate Bayesian principles, enabling probabilistic predictions and uncertainty estimation.

Using simulation and experiment data, the study compares the interval prediction performance of the two proposed BNN models for all coordinates of the end effector positions and orientations vector. The study also discusses the performance of the BNN models in depth, particularly for the *P*_*x*_ coordinate using two datasets: one collected from the entire workspace of the 6-RSU simulator, and the other collected from the workspace except for some regions within it.

The study's experiment results demonstrate that the proposed NARX-BNN outperforms BNN-1 in predicting the FGM of the 6-RSU parallel manipulator. The NARX-BNN reduces the RMSE of the all-predicted values by up to 11 % and reduces the Average Width indicator of the prediction interval by approximately 12.7 %, both at a 95 % confidence level, compared to BNN-1.

These evaluation results demonstrate the robustness of the NARX-BNN as an effective approach for approximating the highly nonlinear geometric system of the 6-RSU parallel manipulator and accurately predicting its uncertainties.

Future investigations may involve the deployment of the proposed model on an actual manipulator and other types of robotic systems. Overall, this study provides a promising approach for accurately predicting the FGM of a 6-RSU parallel manipulator and improving the reliability of machine learning models in robotics.

## CRediT authorship contribution statement

**Alaa Aldeen Joumah:** Writing – review & editing, Writing – original draft, Validation, Software, Project administration. **Assef Jafar:** Supervision. **Chadi Albitar:** Supervision.

## Data availability

Data will be made available on request. Anyone looking for any data can directly contact the corresponding author or first author upon reasonable request.

## Financial Support

This research received no specific grant from any funding agency, commercial or not-for-profit sectors.

## Declaration of competing interest

The authors declare that they have no known competing financial interests or personal relationships that could have appeared to influence the work reported in this paper.
